# Preparation and Optimization of In Situ Gel Loaded with Rosuvastatin-Ellagic Acid Nanotransfersomes to Enhance the Anti-Proliferative Activity

**DOI:** 10.3390/pharmaceutics12030263

**Published:** 2020-03-13

**Authors:** Khaled M. Hosny, Waleed Y. Rizg, Rasha A. Khallaf

**Affiliations:** 1 Department of Pharmaceutics, Faculty of Pharmacy, King Abdulaziz University, Jeddah 21589, Saudi Arabia; bsupharma@gmail.com; 2 Department of Pharmaceutics and Industrial Pharmacy, Faculty of Pharmacy, Beni-Suef University, Beni-Suef 62511, Egypt; malak.diwan2008@gmail.com

**Keywords:** rosuvastatin, antioxidant, ellagic acid, box behnken design, nanotransferosomes, in situ gel, human chondrosarcome-3 cell line, tongue carcinoma

## Abstract

The objective of this study was to develop an optimized sustained-release nanotransfersomes (NTS) based in situ gel formulation of rosuvastatin (RO) combined with ellagic acid (EA) antioxidant, to enhance cytotoxic and anti-proliferative activity against tongue carcinoma. The concentrations of lecithin, Tween 80, and d-tocopherol polyethylene glycol succinate (TPGS) were considered as independent variables. Particle size, entrapment, and stability were selected as dependent variables. The obtained formulation containing 25% lecithin, 20% Tween 80, and TPGS 15% fulfilled the prerequisites of the optimum formulation. RO-NTS loaded in situ gel was prepared and optimized for concentrations of Poloxamer 407, and Carbopol, using statistical design. Drug release from in situ gel showed a sustained release profile. The RO IC50 was decreased by half for the in situ gel in comparison to plain RO and RO-EA-NTS. A significant amount of caspase-3 was detected in all the formulation treatments. The studies indicated that EA’s synergistic anti-oxidant effect owing to a high affinity to the PGP efflux transporter and higher penetration in the RO-NTS formulation led to a higher inhibition against human chondrosarcome-3 cancer cell lines. RO-EA NTS–loaded in situ gel had a sustained release that could be significant in localized therapy as an alternative to surgery in the treatment of aggressive tongue carcinoma.

## 1. Introduction

Tongue cancer is a well-known type of mouth cancer. Like other cancer types, it develops as a result of uncontrolled cell divisions, which, in this case, are the squamous cells present on the tongue surface [1]. Troubled swallowing, painful tongue, and difficulty in healing of sores and lesions are the most noticeable signs of tongue cancer. Different triggers can cause such carcinoma, including viruses like the papillomavirus, in addition to common causes like heavy tobacco smoking, alcoholism, and jagged teeth [2].

Statins are one of the most commonly prescribed drugs all over the world. They are well known for their cholesterol-lowering effect; hence, they are mainly used to treat and protect against cardiovascular diseases [3]. Recently, several investigations have explored the pleiotropic effects of statins that may enable them to treat conditions other than cardiac diseases, such as inflammation, neurological conditions, autoimmune diseases, and even cancer. Many researchers evaluated the combined effects of statins with some anticancer drugs. For example, lovastatin increased the anticancer activity of doxorubicin against colon and breast cancer in mice [4,5]. Additionally, mevastatin increased the sensitivity of lung cancer and human leukemia cell lines to anthracyclines [6,7]. Administration of statins combined with antioxidant compounds of natural origin can yield better results as shown in the combination of simvastatin and alpha-lipoic acid that improved toxicity against breast carcinoma cell lines [8].

Transferosomes, defined as vesicular colloidal dispersions composed mainly of phospholipids and nonionic surfactants acting as edge activators (EA), have attracted a great deal of attention in the field of drug delivery [9]. The presence of such EA weakens the bonds between phospholipids molecules and imparts the property of elasticity onto transferases making them ultra deformable vesicles. Transferosomes have several advantages that make them successful vesicular drug delivery systems, including (a) being biodegradable and biocompatible with human body, and (b) the ability of EA to solubilize and stabilize poorly soluble drugs owing to their surface activity, low toxicity, enhanced tissue targeting capacity, and ability to obtain prolonged drug release [10,11,12]. RO, also known as “superstatin,” is the most effective antihyperlipidemic agent in the statins family. It is more efficient and attains fewer side effects compared to other statins although it exhibits poor solubility and poor bioavailability [13]. Several studies have proven the anticancer activity of RO. It showed cytotoxic effects against thyroid cancer cells in vitro as it increased the caspase-3 activity and apoptosis confirmed by DNA fragmentation analysis [14], and reduced viability in cervical, hepatic, and breast cancer cell lines [15]. Moreover, it was reported to decrease the invasive potential of prostate cancer cells [16]. Such antiproliferative action of RO could be attributed to its ability to down-regulate the expression of the DDX3 gene that plays a crucial role in cancer cell division [14].

Ellagic acid (EA) is a polyphenolic phytocompound abundant in natural sources such as green tea, pomegranate, and blackberries [17]. EA has been reported to have antioxidant and anticarcinogenic activity [18]. A combination of RO and EA can potentiate their anticancer effect.

Gel systems that are applied as drops and transform into a gel at the application site are called in-situ gelling systems. Gelation of such systems can be induced by temperature change, pH change, ions interactions, solvent exchange, or UV induction. In situ gels usually undergo reversible sol-gel phase transitions [19]. In temperature-induced gelation, systems are freely flowing at room temperature and gelling of a solution is triggered by temperature change and, in such cases, they are called thermosensitive gels. Poloxamers, being triblock co-polymers prepared from poly(ethylene oxide)-*b*-poly(propylene oxide)-*b*-poly(ethylene oxide), are the most commonly used thermosetting polymers and could be applicable for the development of effective thermosensitive drug delivery [20].

Poloxamer 407 (P 407) is usually used in concentrations above 20 to 23% in in situ gels; however, it may cause some patient discomfort. A concentration of 16% or lower of P 407 was reported to be safer. To solve this problem, polyvinyl alcohol (PVA) is usually used to induce micelle entanglement and allow lower concentrations of P 407 to be used in the in situ gel formulation [21].

Incorporating vesicular drug delivery systems such as transferosomes into an in situ gel base can allow precise delivery and controlled release of the drug into the targeted site. To our knowledge, the anticancer effect of RO along with EA has not been explored before. Therefore, the goal of the current study is to develop an in situ gel loaded with RO EA based NTS combination to obtain sustained drug release and examine its effectiveness against HSC-3 tongue carcinoma cell lines.

## 2. Materials

Bayer, Germany gifted the Rosuvastatin Calcium. Isopropyl myristate, d-tocopherol polyethylene glycol succinate (TPGS), and egg lecithin were procured from Acros Organics, USA. Tween 80, stearic acid, Poloxamer 407, 188, and polyvinyl alcohol (PVA) (80% hydrolyzed; Mw: 9000–10,000) were purchased from Sigma Aldrich, St. Louis, MO., USA. King Abdul-Aziz University, Saudi Arabia, gifted human chondrosarcome-3 cell lines. All additional chemicals, reagents, and solvents used were of analytical grade.

## 3. Methods

### 3.1. Preparation of RO-EA Based NTS

RO-EA based NTS were prepared by the conventional thin-layer evaporation technique [22] using factorial design. A 3-factor three-level Box–Behnken design was employed to study the effect of independent variables on dependent variables as shown in Table 1. A total of 17 formulations was prepared according to the experimental design. In a dry, round-bottom flask containing a solvent mixture (chloroform: methanol (2:1, *v*/*v*), phospholipids, surfactant (edge activator), and drugs were dissolved. A thin lipid film was formed while the organic mixture was evaporated at 60 rpm, at 45 °C using a vacuum rotary evaporator (Heidolph rotavap (P/N Hei-AP Precision ML/G3, Schwabach, Germany). Solvent residues were removed overnight using a desiccator. Furthermore, lipid vesicles were formed by slight hydration of the thin lipid film using phosphate-buffered saline (pH 7.4) at room temperature. Unilamellar lipid vesicles were obtained by sonication (Branson, Danbury, CT, USA) of the formulation for 15 min. The final formulations were collected and stored at 4 °C for further characterizations.

#### 3.1.1. Experimental Design

Nanotransferosomal gel of FT was prepared using the Box–Behnken factorial design, where a three-factor and three-level design was employed to study the relationship between selected independent variables and dependent variables (Table 1). This method was performed using Design Expert 10 (Stat-Ease Inc., Minneapolis, MN, USA). Response surface graphs were used to appraise the factor association between the variables. The selected independent variables considered were the concentration of egg lecithin (%) (X_1_); Tween 80 (%) (X_2_); and TPGS (%) (X_3_), while the three factorial levels for these variables were coded as −1, 0, and +1 for low, medium, and high levels, correspondingly [23]. Particle size (PS-Y_1_), entrapment efficacy (EE-Y_2_), and the stability index (Y_3_) were selected as the dependent variables. Seventeen formulations were required to analyze the interaction of each level on formulation characters. ANOVA was used to establish the statistical validity of the polynomial equations generated. All responses observed were fitted concurrently to different models. The best-fitting experimental model (main, interaction, and quadratic) was taken statistically based on a comparison of several statistical parameters like the CV (coefficient of variation), R^2^ (multiple correlation coefficient), adjusted R^2^ (Adju.R^2^), and the predicted R^2^ (Pred.R^2^). The level of significance was considered at *p*-value < 0.05. The non-linear quadratic design matrix is defined below,

Y_i (Quadratic)_ = b_0_ + b_1_X_1_ + b_2_X_2_ + b_3_X_3_ + b_12_X_1_X_2_ + b_13_X_1_X_3_ + b_23_X_2_X_3_ + b_11_X_1_^2^ + b_22_X_2_^2^ + b_33_X_3_^2^ (1)

where Yi—dependent variable; b_0_—arithmetic mean response of all trials; b_1_, b_2_, b_3_ are linear coefficients; b_12_, b_13_, b_23_ are interaction coefficients; b_11_, b_22_, b_33_ are quadratic coefficients; X_1,_ X_2,,_ and X_3_ (main effects)—average value of changing factor one at a time, X_1_X_2_ and X_1_X_3_ and X_2_X_3_—represent the interaction terms; and X_1_^2^, X_2_^2^, and X_3_^2^—the polynomial terms.

#### 3.1.2. Characterization of NTS Formulations

##### Vesicles Shape, and Size Distribution

Prepared RO-EA-NTS formulations were characterized for vesicle shape using a scanning electron microscope (Morgagni 268D-Fei Electron Optics, Eindhoven, the Netherlands) [24]. Image capture and analysis were performed using a digital micrograph and soft imaging viewer. The vesicle’s size distribution was determined using a computerized inspection system, Malvern Instruments (Malvern, UK), by a dynamic light scattering process [25].

##### Determination of Zeta Potential

Zeta potential was measured using a Malvern Zetasizer instrument (#Nano ZS 4800, Malvern, UK) by performing DLS (dynamic light scattering) experiments at 25 °C, with a constant scattering angle of 173°. Each measurement was done three times (*n* = 3) and the average value was calculated [26].

##### Entrapment Efficiency

EE was expressed in terms of the percentage of the total amount of drugs found in the prepared formulations, and this was done using an indirect method. The unentrapped drug dissolved in the solvent could be quantified and differentiated, helping to estimate the actual drug entrapped within the vesicles. Prepared RO-EA-NTS loaded in situ gel was taken into a Petri dish and freeze-dried. To the known amount of dry sample, acetonitrile was added and shaken thoroughly [27]. This sample was centrifuged at 10,000 rpm for about 1 h and the supernatant liquid was collected. Further washings were done with acetonitrile and all the washings were collected. Supernatant liquid and all the washing contents were mixed and dried on a water bath. To this dried extract, methanol was added and diluted. Absorbance was read at 450 nm for RO. EE was calculated using the following formula,

EE (%) = [C_total_ − C_free_] / C_total_ × 100 (2)

where, C_total_ is the theoretical amount of RT. C_free_ is the amount of drug detected in the supernatant.

##### Surface Morphology

The surface morphology of the prepared formulations was characterized using a scanning electron microscope (SEM). The sample was reconstituted with deionized water. The sample was spread over the carbon tape, dried, coated with gold, and examined under SEM [28].

##### Stability Studies/Stability Index

Stability studies were performed for optimum formulation for future commercial practicability. The formulation was filled into an amber-colored glass bottle with a screw cap and stored at 4.0 ± 1 °C and 25 ± 2 °C for a period 30 days. Samples were withdrawn at intervals and evaluated for particle size and residual drug content. Results were analyzed using ANOVA which shows significant or nonsignificant differences (*p* < 0.05) in the results before and after storage.

### 3.2. Preparation of RO-NTS–Loaded In Situ Gel

RO-EA-NTS in situ gel was prepared using the Box–Behnken factorial design, where a two-factor and three-level design was employed to study the relationship between selected independent variables and dependent variables (Table 2). In a required quantity of deionized cold water, P 407 and methylparaben were added and solubilized. An appropriate quantity of prepared RO NTS was added to the above polymer solution. Stirring was continued until the uniform solution was obtained. The formed solution was jellified by adding a prescribed quantity of Carbopol 934 P under continuous stirring using a mechanical stirrer at 500–1000 rpm. The final preparation was kept under refrigeration at 5 °C for about 10–12 h. This helps in the complete dissolving of polymers in the solution. Finally, the pH of the solution was adjusted by adding a small amount of triethanolamine (TEA). Carbopol 934 P and P 407 were added in concentrations outlined in the experimental design portrayed by the design expert [2].

#### 3.2.1. Determination of Gelation Temperature

A modified Miller and Donovan technique [29] was used to assess the gelation temperature. Test tubes containing around 2 mL of aliquots of the sample were sealed with parafilm and then immersed in a water bath, maintained at a temperature of 4 °C. Then the bath temperature was increased slowly at increments of 1 °C and left for 15 min to equilibrate at every new setting. The samples were examined for gelation temperature, where the meniscus was static on slanting by 90 °C [30].

#### 3.2.2. Determination of Viscosity

The optimized formulations were taken in a beaker and we measured the viscosity using a Brookfield viscometer (LVDV III U, Brookfield Engineering Labs, Stoughton, MA, USA). The measurements were done 10 min after the dipping of spindle no. 62, which was rotating at a speed of 50 rpm [31,32]. Viscosity was measured before and after the gelation of the formulation.

#### 3.2.3. In-Vitro Release Studies

RO release from the RO-EA NTS–loaded in situ gel and plain RO-EA–loaded in situ gel was determined using a membrane-less dissolutions model. 1 g of prepared formulation (in the cold state) was placed in a glass tube and put in a water bath of 37 °C. After the gelation of the formulation, 500 µL of pre-equilibrated phosphate buffer (pH 7.2) at 37 °C was added. At predetermined intervals of time (at 0, 2, 8, 24, 48, 72, 96, 144, 168, and 192 h), the complete medium was aliquoted and analyzed at λ_max_ 304 nm in triplicate using the formerly reported HPLC method. After sampling, the medium was replaced with an equal amount of fresh medium [33].

#### 3.2.4. In Vitro Cell Viability Assay

In vitro cytotoxicity of optimized formulations of RO-EA NTS, RO-EA NTS–loaded in situ gel, and plain RO was evaluated against human chondrosarcome-3 cells, using the 3-[4,5-dimethylthiazol-2-yl]-2,5-diphenyltetrazolium bromide (MTT) method. Human chondrosarcome-3 cells of density of 5 × 10^4^ cells/mL were trypsinized using 0.25% trypsin-EDTA and added to a 96-well plate (approximately 2500–5000 cells/well). After 1 day, the total medium was replaced with DMEM (Dulbecco’s Modified Eagle Medium). The control cells were treated with 5-fluorouracil and the remaining cells were treated with plain RO, RO-EA NTS, and RO-EA NTS–loaded in situ gels at a concentration of 10 to 50 μg/mL. After incubation for about 72 h, 0.1 mL of DMEM having 0.2 mg/mL MTT was added and incubated for 2–3 h. DMEM was replaced with DMSO to dissolve the formed formazan. Then the absorbance was measured using a microplate reader (Biotek Synergy, Salt Lake City, UT, USA) at 540 nm. Finally, the dose-responsive curve was used to determine the IC 50 values [34].

#### 3.2.5. Caspase-3 Enzyme Assay

The caspase-3 enzyme assay was performed for plain RO, RO-EA NTS, and RO-EA NTS–loaded in situ gels. Initially, the cells were cultured in ROMI 1640 (containing 10% fetal bovine serum at 37 °C) and cell extraction buffer was used for further lysis. The collected lysate was diluted with standard buffer as per the assay range for human active caspase-3 traces, and the cells were plated in DMEM (100 μL) at (1.3–1.9) × 10,000 cells/well. Each sample was inoculated in a 96-well plate, 24 h before the caspase- assay [35]. The assay was carried out (as per the kit instructions-USCN Life Science Inc., Nanjing, China) using spectrophotometry at 450 nm.

#### 3.2.6. Statistical Analysis

Data were analyzed for multiple comparisons using a one-way analysis of variance (GraphPad Prism v5.01, GraphPad Software, San Diego, CA, California) and the level of significance was set at *p* < 0.05.

## 4. Results

### 4.1. Preparation of NTS

From the Box–Behnken factorial design, a total of 17 runs were projected by the design expert for three factors: egg lecithin (%) (X_1_); Tween 80 (%) (X_2_); and TPGS (%) (X_3_), which were varied at three different levels (coded as −1, 0, and +1). Particle size (PS-Y_1_), entrapment efficacy (EE-Y_2_), and the stability index (Y_3_) were studied as responses. The observed values of the responses are shown in Table 3. The obtained particle size was found to be in the range of 75 to 167 nm, the calculated EE was about 28% to 85%, while the stability index was estimated at around 41 to 91.1%. Statistical analysis of the selected variables on the responses was studied using the Design Expert® 12.03.0 (Stat-Ease, Inc., Minneapolis, MN, USA).

**Figure 1:**
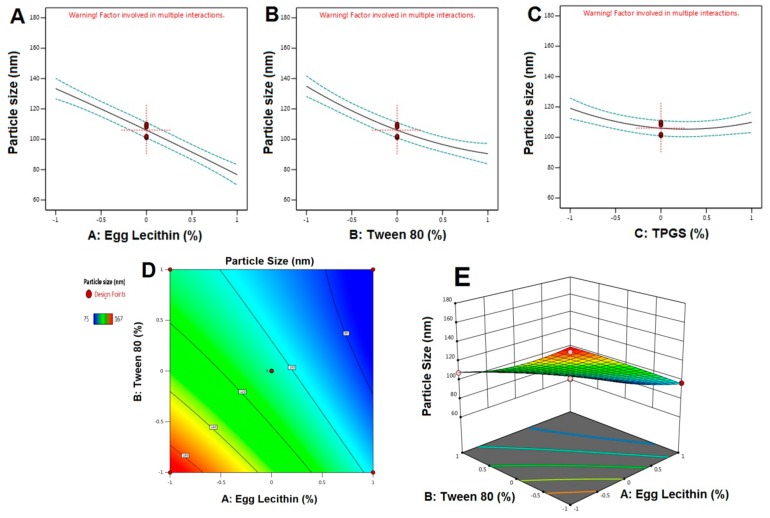
Main effect diagram, contour, and 3-D response surface plots representing the effect of the studied variables on particle size (Y1). (**A**). Main effect diagram of Egg Lecithin (%) on particle size; (**B**). Main effect diagram of Tween 80 (%) on particle size; (**C**). Main effect diagram of TPGS (%) on particle size; (**D**). Contour Plot showing the effect of Egg lecithin and Tween 80 on Particle size; (**E**). 3-D Surface plot representing the effects of the egg lecithin (%) and tween 80 (%) on particle size.)

**Figure 2:**
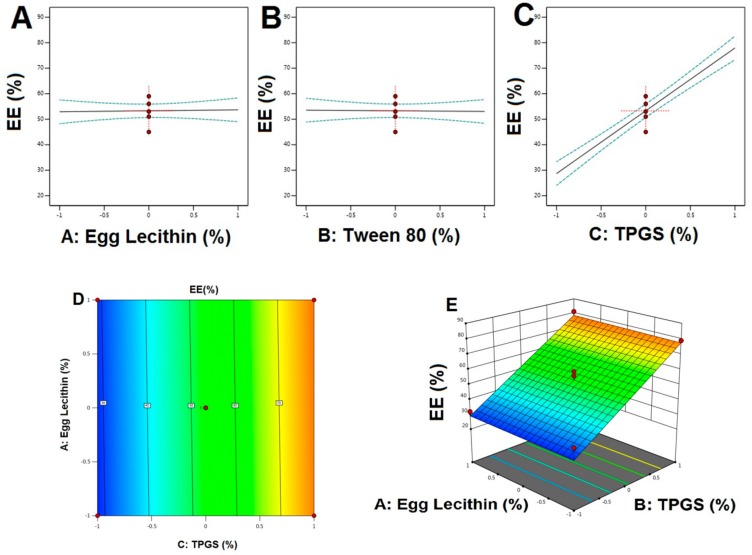
Main effect diagram, contour, and 3-D response surface plots representing the effect of studied variables on the EE (Y2). (**A**). Main effect diagram of Egg Lecithin (%) on EE%; (**B**). Main effect diagram of Tween 80 (%) on EE%; (**C**). Main effect diagram of TPGS (%) on EE%; (**D**). Contour Plot showing the effect of Egg lecithin and TPGS on EE; (**E**). 3-D Surface plot representing the effects of the egg lecithin (%) and TPGS (%) on EE.

**Figure 3:**
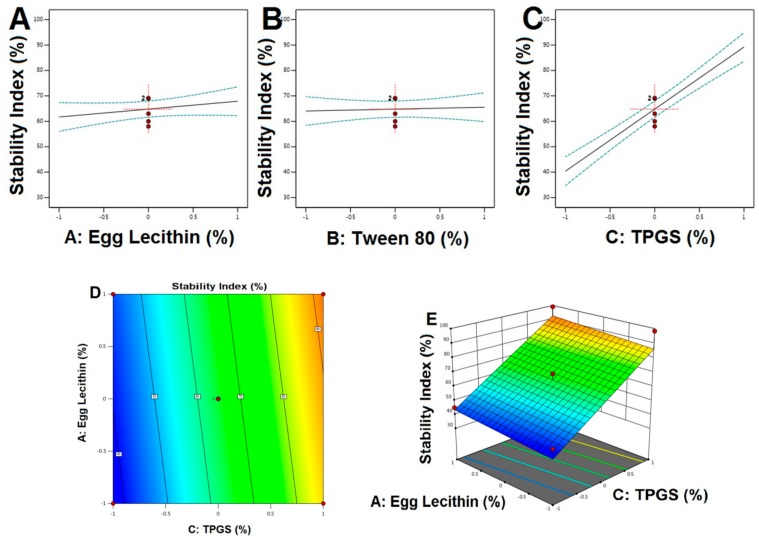
Main effect diagram, contour, and 3-D response surface plots representing the effect of studied variables on the stability index (Y3). (**A**). Main effect diagram of Egg Lecithin (%) on stability index; (**B**). Main effect diagram of Tween 80 (%) on stability index; (**C**). Main effect diagram of TPGS (%) on stability index; (**D**). Contour Plot showing the effect of Egg lecithin and TPGS on stability index; (**E**). 3-D Surface plot representing the effects of the egg lecithin (%) and TPGS (%) on stability index.

Calculated F values, p values, and estimated effects for the particle size, EE, and stability index are depicted in Table 4. Figure 1, Figure 2 and Figure 3 contain the interaction of selected responses with the variables, contour plot, which confirms the effect of the variables. The derived equations of the responses for the best fit model were given as follows.

Particle size = +106.00 − 28.37A − 22.25B − 4.63C + 9.25AB + 2.50AC + 0.2500BC −1.0000A^2^ + 6.75B^2^ + 8.50C^2^ (3)

EE = +53.29 + 0.3750A − 0.2500B + 24.63C(4)

Stability index = +64.79 + 3.10A + 0.7500B + 24.43C(5)

**Figure 4:**
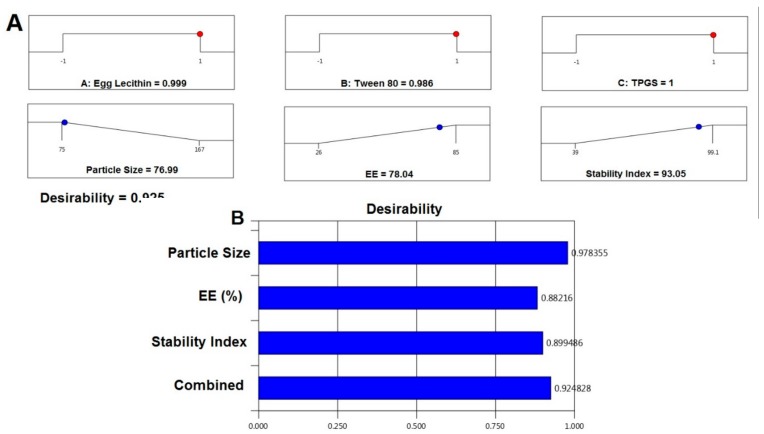
Desirability ramp and bar chart for optimization. (**A**). Desirability ramp shows the levels for independent variables and predicted values for the responses of the optimum formula; (**B**). Bar Chart shows the desirability values for the combined responses.

A, B, C, and AB interaction terms affected the particle size and only the C term influenced the residual responses (EE and stability index). The optimized RO NTS formulation was selected based on criteria to attain minimum particle size, maximum EE, and maximum SI, using all the variables within the range as shown in Table 5. The global desirability function (D) was applied simultaneously to optimize the series of models obtained from experimental statistical analysis. All three independent variables were incorporated in the optimization at their design space. The desirability plot for the responses showed a maximum D value of 0.925 that was obtained at optimum concentrations of the independent variables. Based on this desirability approach, a formulation containing 1% of egg lecithin, 0.998% of Tween 80, and TPGS of 1% could fulfill the prerequisites of optimum formulation (Figure 4).

### 4.2. Preparation of RO-EA NTS Loaded In Situ Gel

RO-NTS loaded in situ gel was prepared using P 407 and Carbopol 934 P at different concentrations, as shown in Table 6. The effect of these variables was evaluated mathematically using ANOVA and regression equations. The formulation of the in situ gel was optimized by considering viscosity before (Y1) and after gelation (Y2) and gelation temperature (Y3) as responses. A total of 13 runs were carried out and the results are shown in Table 6. Viscosity by centipoises (Cp) before gelation was found to be in the range of 1.15 ± 0.45 to 4.06 ± 0.14 Cp and gelation temperature was about 20–38 °C. After gelation, Viscosity was increased drastically and found to be in the range of 8.51 ± 0.13 to 29.86 ± 0.51 Cp. The formulation was optimized by selecting the responses (Y1, Y2, and Y3) with the minimum, maximum, and maximum criteria.

**Figure 5:**
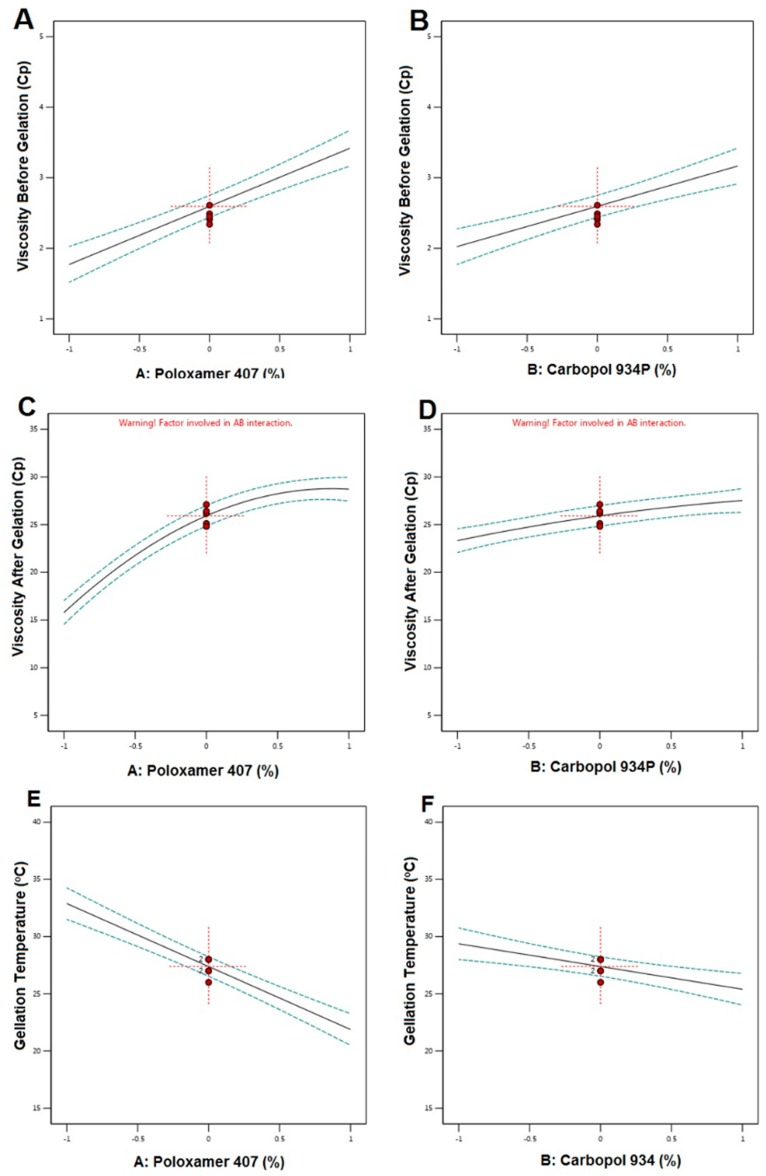
Main effect diagram representing the effect of studied variables on viscosity before gelation (Y1), viscosity after gelation (Y2), and gelation temperature (Y3). (**A**). Main effect plot showing the effect of Poloxamer 407 % on the viscosity before gelation; (**B**). Main effect plot showing the effect of Carbopol 934% on the viscosity before gelation; (**C**). Main effect plot showing the effect of Poloxamer 407 % on the viscosity after gelation; (**D**). Main effect plot showing the effect of Carbopol 934% on the viscosity after gelation; (**E**). Main effect plot showing the effect of Poloxamer 407 % on the gelation temperature. (**F**). Main effect plot showing the effect of Carbopol 934% on the gelation temperature.

The interaction of selected responses and contour plots was shown in Figure 5. Calculated F values, p values, and estimated effects for the Y1, Y2, and Y3 are depicted in Table 7. Regression equations for the best fit model for all the responses were as follows:

Viscosity Before Gelation = +2.59 +0.8240A +0.5717B(6)

Viscosity After Gelation = +25.92 +6.47A +2.10B +0.1950 B − 3.68A^2^ − 0.4964B^2^ (7)

Gelation Temperature = +27.38 − 5.51A − 1.99B(8)

**Figure 6:**
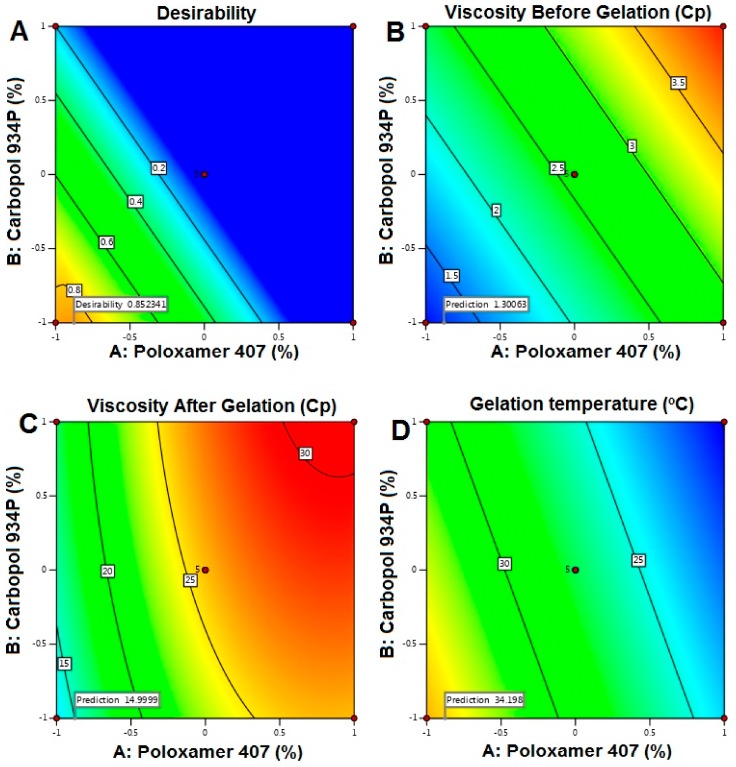
Contour plots representing the effect of studied variables on selected responses and desirability. (**A**). Contour plot representing the effect of studied variables on desirability; (**B**). Contour plot representing the effect of studied variables on viscosity before gelation; (**C**). Contour plot representing the effect of studied variables on viscosity after gelation; (**D**). Contour plot representing the effect of studied variables on gelation temperature.

The desirability plot for the responses showed a maximum D value of 0.852 that was obtained at optimum concentrations of independent variables. Based on this desirability approach, a formulation containing Poloxamer 407-7% and Carbopol 934P 0.2% could fulfill the prerequisites of optimum formulation (Figure 6).

### 4.3. Characterization of NTS Formulations

**Figure 7:**
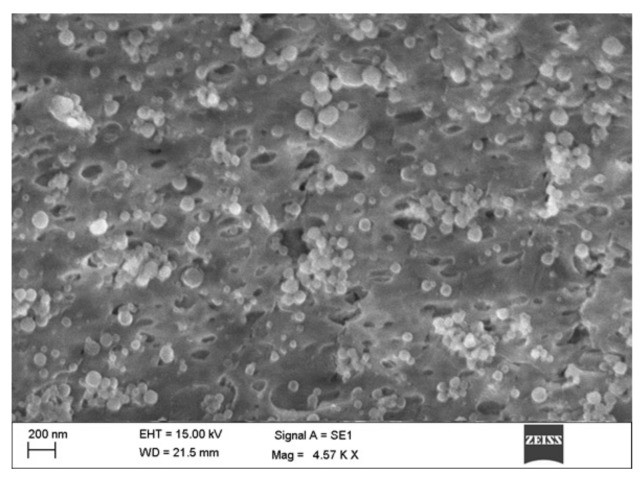
Scanning electron microscope (SEM) image of NTS–loaded in situ gel.

RO EA based NTS were characterized for vesicle shape using a scanning electron microscope. The surface morphology of the prepared formulations was found to be almost spherical with loose aggregates (Figure 7). The vesicle size distribution and zeta potential were determined using a dynamic light scattering process. Vesicle size was maintained uniformly and zeta potential for the optimum formulation was found to be 167 nm and 30.87 mV, respectively.

### 4.4. In-Vitro Release Studies

**Figure 8:**
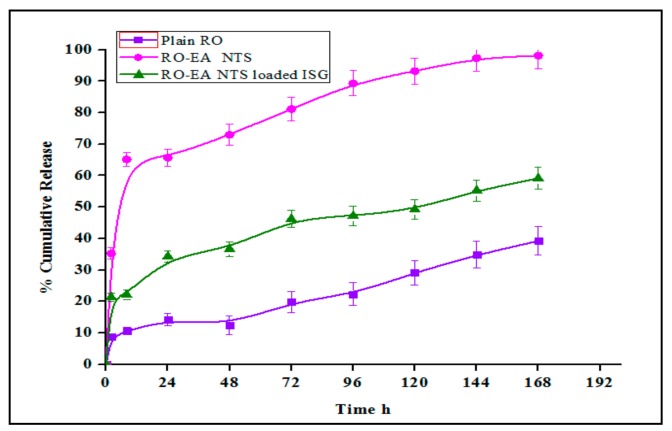
Comparative in-vitro release profiles of plain RO aqueous dispersion, RO-EA-NTS aqueous dispersion, and RO-EA-NTS loaded in situ gel formulations.

RO release from the RO-EA, NTS–loaded in situ gel, and the plain RO-EA–loaded in situ gel, was determined using a membrane-less dissolutions model and the results were shown in Figure 8. Studies were carried out up to 168 h.

### 4.5. Stability Studies

Stability studies were performed as per Q1A (R2) of the ICH guidelines using RO-EZ NTS and RO-EA NTS in situ gel, which were stored at 4 ± 3 °C and 25 ± 2 °C. Particle size and residual drug content were determined at frequent intervals of 15 days and 30 days as shown in Table 8.

### 4.6. In Vitro Cell Viability Assay

**Figure 9:**
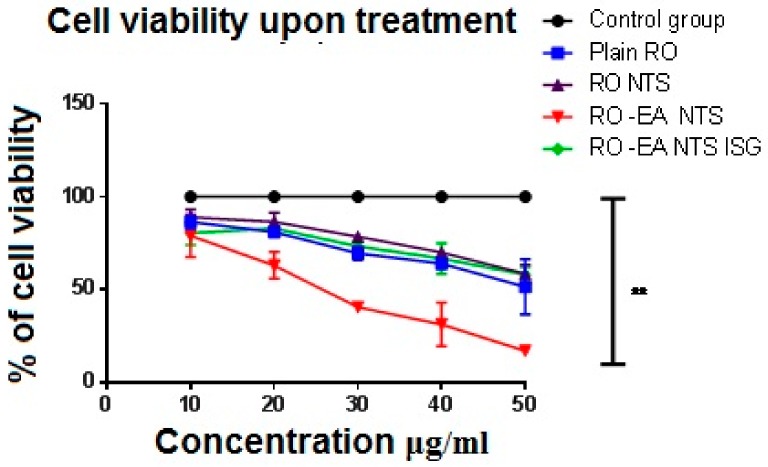
Effect of plain RO, RO-NTS, RO-EA-NTS, and RO-EA-NTS loaded in situ gel, and 5 Fluoro-Uracil (control) on the viability of the HSC-3 cell line. The values represent the mean ± SD of three independent experiments (*n* = 9).

The MTT method was used to evaluate in vitro cytotoxicity against human chondrosarcome-3 cells for optimized formulations of RO-EA NTS, RO-EA NTS–loaded in situ gel, and plain RO after 72 h of treatment. Figure 9 shows the comparison of percentage cell viability of the prepared formulation, where cell viability was normalized to 100 for the control group.

### 4.7. Caspase-3 Enzyme Assay

**Figure 10:**
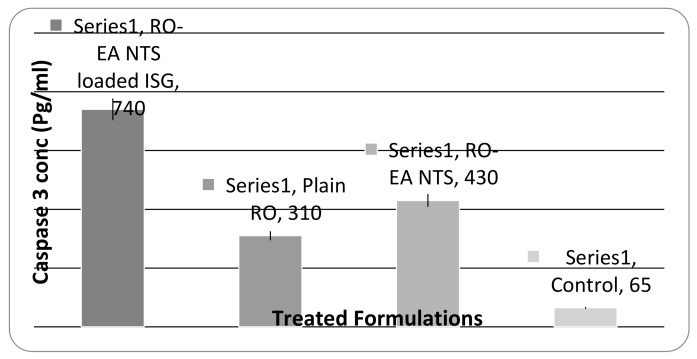
Caspase-3 enzyme concentrations in human chondrosarcome-3 cells treated with different formulations, and control (solvent-free); data are presented as the mean ± SD. (*n* = 6).

The caspase-3 enzyme assay was performed by inoculating the samples in 96 well plates. A significant amount of caspase-3 was detected in the human chondrosarcome-3 cells in all the treatment formulations, as depicted in Figure 10.

## 5. Discussion

### 5.1. Formulation Optimization of RO EA Based NTS

Statistical analysis was performed for the inference of the quantitative effects of the fact factors. The recommended considerable model was quadratic for PS, where the R^2^ value was found to be 0.9689; while the 2FI had R^2^ values of 0.9219 and 0.8882 suggested for EE and stability index responses. Values of *p* < 0.05 indicate that model terms are significant. A positive value indicates a synergistic effect, while a negative sign represents an inverse effect [36]. 

For particle size, based on the ANOVA results (Table 4), both the F and p values were used to measure the significance of the coefficients of the model. PS showed that a signal to noise ratio of 7.765, indicating high adequacy of the selected model. From the regression equation, it confirmed that response Y_1_ (PS) was influenced significantly by (i) the antagonistic effect of A, B (with a probability value of <0.001), and C (0.0285) and (ii) the synergistic effect of AB with a *p*-value of 0.0060, being that A effects were the highest. Change in the particle size of transferosomes was primarily due to that a small change or increase in the concentration of TPGS may result in the formation of particles with the desired or minimal size. Variation in the concentration of TPGS will also result in the enhanced entrapment efficiency. In contrast, the only concentration of TPGS (with a probability value of <0.001) was synergistically affecting the EE%. This result could be due to the Tween 80 or because of the lipophilic nature of the drug, which could enhance the partitioning into the vesicles. The model F-values for the EE and stability index were found to be 63.96 and 43.39, which implied that the model was significant. There was only a 0.01% chance that an F-value this large could occur due to noise. Optimization of the independent variables was done by setting goals for each response then creating an overlay graph [37]. The PS response was set to a minimum goal, while the EE and stability index was adjusted to maximize. Thus, an individual desirability function (D) was proposed for each response where the values were expressed using a non-dimensional scale ranging in the interval 0–1. The desirability plot for both the responses showed a maximum D value of 0.925, which was obtained at optimum concentrations of independent variables. Accordingly, the use of such conditions would attain a small PS with a maximum EE and stability index. Consequently, the optimum formulation was prepared by considering the optimum concentrations of the variables, and it was evaluated for PS, EE%, and the stability index. Experimental values were found to be following the theoretical values [38].

### 5.2. Preparation of RO-NTS Loaded In Situ Gel

Viscosity Before Gelation = +2.59 + 0.8240A + 0.5717 B(9)

Viscosity After Gelation= +25.92 + 6.47A + 2.10B + 0.1950AB − 3.68A^2^ − 0.4964B^2^ (10)

Gelation Temperature = +27.38 - 5.51A − 1.99B(11)

The 2FI was recommended for the Y1 and Y3 responses, where the R^2^ value was found to be at a maximum (0.9115 and 0.9221), and the quadratic model was suggested for Y2 where the R^2^ value was found to be 0.9736. Based on the ANOVA results, response Y1 was affected significantly (*p*-value < 0.0001). The model F-value of 62.80 implied that the model was significant. There was only a 0.01% chance that an F-value this large could occur due to noise. Both the selected responses had almost the same magnitude of the synergistic effect on Y1. There would be an increase in the viscosity before gelation with respect to an increase in the concentrations of P 407 and Carbopol 934 P. From the regression equation of Y2; it confirmed that the response was significantly influenced by (i) the synergistic effect of A, B with a *p*-value of <0.0001, 0.0006, being that Poloxamer 407 % effect was the highest and (ii) the antagonistic effect of A^2^ (with a probability value of <0.001) [39]. Gelation temperature was significantly affected by both the selected factors but in an antagonistic way, where the concentration of P 407 was more effective than Carbopol. There would be a decrease in gelation temperature due to an increase in these factors, and this might be due to the tightly-packed gel structure arrangement in the lattice pattern.

Each response was set for the goals, and the desirability plot for both responses showed a maximum D value of 0.852, which was obtained at optimum concentrations of the independent variables [40,41]. Accordingly, the use of such conditions would achieve the required viscosity before and after gelation with less gelation temperature. Moreover, the optimum formulation was prepared by considering the optimum concentrations of the variables evaluated.

### 5.3. SEM, Average Particle Size, and Zeta Potential

Surface morphology studies justify the vesicular characteristics with lamellae of the vesicles. They appeared as unilamellar vesicles and were distributed uniformly without any aggregation. Fully saturated polymer and a minimal solvent diffusion rate led to the formation of smooth, spherical, and homogenously distributed particles. Optimized NTS formulation was found to have an average diameter of 76.32 ± 0.0312 nm, which confirmed the monodispersity, thereby indicating mono-dispersity of the formulation [42]. A high potential of (>30 mV and <−30 mV) is considered as favorable for good stability of the prepared dispersions. Zeta potential for the optimum formulation was found to be +30.87 mV, which ensured high stability.

### 5.4. Stability Studies

Stability studies performed on the RO-EA NTS and RO-EA NTS in situ gel, for particle size and residual drug content, before and after storage, were determined using the same methods given earlier, and at frequent intervals of 15 and 30 days (Table 8). Percentage residual drug content was determined by considering an initial drug content of 100% and it showed that 0.91%–2.10% of the drug was lost from the formulation at the end of 30 days when stored at 4.0 ± 1 °C. Approximately 3.9% of the drug was lost from the formulations when stored at 25 ± 2 °C. The effect might be due to the leaching of the drug from the prepared NTS at higher temperatures. For particle size, there was no significant change when stored at 4.0 ± 1 °C, but there was a slight change when stored at 25 ± 2 °C. From these studies, we could confirm that there was better stability of the formulation under refrigerated conditions.

### 5.5. In Vitro Drug Release, Cell Viability Studies, and Caspase-3 Enzyme Assay

The cumulative percentage drug release from plain RO, RO-EA NTS, and RO-EA NTS loaded in situ gels was studied as a function of time. At 2 h, an initial burst release was observed from RO-EA NTS, but at the end of 8 h, 65.14% of the drug was released in contrast to plain RO (10.61%) and RO-EA NTS loaded in situ gels (22.1%). At the end of 24 h, about 65.68% drug was released from RO-EA NTS, which was reduced to 34.3% for RO-EA NTS loaded in situ gel. The drug release from plain RO was very low because of solubility issues. Drug release from RO-EA NTS loaded in situ gel was slower to plateau up to 96 h but the release continued even after 168 h. Nonetheless, there was 98.1% of cumulative drug release from RO-EA NTS by the end of 168 h, in contrast to the RO-EA NTS loaded in situ gel, which had a steady-state release of 59.1% and could still control the drug release even beyond 168 h. The effect was mainly due to the gelling action of P 407 and Carbopol, which were responsible for the controlled release of the drug. The drug release of RO from the formulations was concentration-dependent, but the drug release from RO-EA NTS loaded in situ gel occurred because of diffusion of RO through the water channels of the gel matrix [43]. The initial burst release of RO from NTS was mainly because of RO presence at the surface of the NTS, which allowed greater diffusion of water through the liquid matrix and was responsible for the faster drug release.

Total four treatments (plain RO, RO-EA NTS, and RO-EA NTS loaded in situ gels) at different concentrations (10–50 µg/mL) were studied for cell viability along with one control group [44]. Figure 9 confirms that all the treatments reduced cell viability in relation to the dose (i.e., in a dose-dependent way). The observed IC50 for RO-EA NTS was lower than that of plain RO and RO NTS, indicating the effect of yielding higher cytotoxicity against HSC-3 cells. There was a significant difference in the IC50 of RO-EA and RO-EA NTS owing to a high affinity to the PGP efflux transporter and higher penetration. Both the caspase-3 enzyme assay and cell viability studies confirmed that the occurrence of apoptosis with RO-EA NTS loaded in situ gel increased considerably in comparison to other treatments. EA was found to enhance the Bax/Bcl-2 ratio, which in turn could activate the caspase-3-mediated apoptosis. Liu et al. confirmed the efficiency of EA in increasing the levels of P21 protein (tumor suppressor protein) and minimizing the levels of anti-apoptotic proteins [45]. There would be a synergistic effect of EA on cytotoxicity and particle size would also have an impact on cellular cytotoxicity [46].

## 6. Conclusions

A novel in-situ gel of rosuvastatin ellagic acid-based nanotransferosomes was formulated and optimized. Based on the desirability approach, a formulation containing Poloxamer 407-7% and Carbopol 934P-0.2% could fulfill the prerequisites of optimum formulation for preparing an RO-EA NTS loaded in situ gel. PDI 76.32 ± 0.0312 nm, confirmed the monodispersity of the formulation. There was better stability of the formulation under refrigerated conditions, as shown in the stability studies. EA loaded formulations showed a considerable increase in apoptosis occurrence through caspase-3-mediation and they also enhanced the tumor suppressor protein levels. EA’s synergistic anti-oxidant effect and higher penetration in the RO-NTS formulation resulted in higher inhibition against human chondrosarcome-3 cancer cell lines. Finally, RO-EA NTS loaded in situ gel may provide a novel strategy (using the deformable vesicular system as carriers) to improve bioavailability, enhance caspase-3-mediated apoptosis, and increase the levels of P21 protein (tumor suppressor protein) in treating tongue carcinomas. This work can still extend to study the in vivo behavior in cancer animal models and permeability studies in cancer tissues.

## Figures and Tables

**Table 1:** Formulation parameters for RO-NTS.

Independent Variables	Levels			Dependent Variables	Constraints
	−1	0	+1		
Concentration of Egg lecithin (%)-X_1_	0	10	25	Particle Size (nm)	Minimum
Concentration of Tween 80 (%)-X_2_	0	10	20	EE (%)	Maximum
Concentration of TPGS (%)-X_3_	0	10	15	Stability Index (%)	Maximum

**Table 2:** Formulation parameters for RO-EA-NTS in situ gel.

Independent Variables	Levels			Dependent Variables	Constraints
	−1	0	+1		
X_1_: P 407 (% *w*/*v*)	05	15	20	Y1: Viscosity before gelation (Cp *)	Minimum
X_2_: Carbopol 934P (% *w*/*v*)	0.2	0.5	1.0	Y2: Viscosity after gelation (Cp)	Maximum
				Y3: Gelation temperature (^o^C)	Maximum

* Cp = Centipoise.

**Table 3:** NTS (nanotransfersomes) formulation compositions with their observed responses.

Run	Factor 1 X_1_: Egg Lecithin %	Factor 2 X_2_: Tween 80 %	Factor 3 TPGS %	Response Y1 Particle Size (nm)	Response Y2 EE %	Response Y3 Stability index %
1	0	−1	1	141	85	85
2	−1	0	1	137	79	98.3
3	0	0	0	110	51	63
4	1	0	−1	85	32	45
5	0	1	−1	101	28	41
6	−1	1	0	108	49	58
7	−1	0	−1	152	36	44
8	0	0	0	102	59	69
9	0	0	0	109	56	69.1
10	0	1	1	93	74	82
11	−1	−1	0	167	49	55
12	1	0	1	80	81	99.1
13	0	0	0	101	45	58
14	0	−1	−1	150	26	39
15	1	−1	0	97	48	66
16	0	0	0	108	53	60
17	1	1	0	75	55	70

**Table 4:** Independent factors for the estimated effects, F-ratio, and associated *p*-values for the NTS.

Factors	PD		EE		SI	
	F-Value	*p*-Value	F-Value	*p*-Value		
Model	56.34	<0.0001 *	43.39	<0.0001	43.39	<0.0001
A-Egg lecithin	284.92	<0.0001 *	2.06	0.1747	2.06	0.1747
B-Tween 80	175.19	<0.0001 *	0.1207	0.7339	0.1207	0.7339
C-TPGS	7.57	0.0285 *	127.99	<0.0001*	127.99	<0.0001 *
AB	15.14	0.0060 *				
AC	1.11	0.3279				
BC	0.0111	0.9192				
A^2^	0.1862	0.6790				
B^2^	8.49	0.0226				
C^2^	13.46	0.0080 *				

Note: * Indicates the significant effect of factors on responses.

**Table 5:** Criteria for the selection of optimized formulation.

Name	Goal	Lower Limit	Upper Limit
A: Egg lecithin	is in range	−1	1
B: Tween 80	is in range	−1	1
C: TPGS	is in range	−1	1
Particle size	minimize	75	167
EE	maximize	26	85
Stability index	maximize	39	99.1

**Table 6:** RO-EA NTS loaded in situ gel formulation compositions with their observed responses.

Run	Factor 1	Factor 2	Response 1	Response 2	Response 3
	A: Poloxamer 407	B: Carbopol 934P	Viscosity Before Gelation	Viscosity After Gelation	Gelation Temperature
	%	%	Cp	Cp	^o^C
1	20	0.5	3.82 ± 0.12	28.14 ± 0.12	21
2	20	0.2	3.01 ± 0.19	26.12 ± 0.11	24
3	15	0.5	2.49 ± 0.38	26.17 ± 0.15	28
4	15	0.5	2.61 ± 0.11	27.1 ± 0.77	26
5	15	0.5	2.41 ± 0.36	25.12 ± 0.11	27
6	5	0.2	1.32 ± 0.87	14.51 ± 0.47	33
7	15	0	1.89 ± 0.22	21.12 ± 0.17	30
8	20	1	4.06 ± 0.14	29.86 ± 0.51	20
9	15	0.5	2.45 ± 0.55	26.41 ± 0.78	27
10	15	1.5	3.25 ± 0.47	28.26 ± 0.77	23
11	5	1	2.93 ± 0.71	17.47 ± 0.89	31
12	15	0.5	2.34 ± 0.78	24.81 ± 0.63	28
13	0	0.5	1.15 ± 0.45	8.51 ± 0.13	38

**Table 7:** Independent factors for the estimated effects, F-ratio, and associated *p*-values for NTS in situ gel.

Factors	Y1		Y2		Y3	
	F-value	*p*-Value	F-value	*p*-Value	F-value	*p*-Value
Model	62.80	<0.0001	89.48	<0.0001	72.05	<0.0001
A-Poloxamer 407	84.79	<0.0001	322.58	<0.0001	127.48	<0.0001
B-Carbopol 934P	40.81	<0.0001	33.97	0.0006	16.61	0.0022
AB			0.1465	0.7133		
A^2^			90.69	<0.0001		
B^2^			1.65	0.2397		

**Table 8:** Results of stability studies for RO-EA NTS and RO- EA NTS in situ gel at different storage conditions.

Formulation		Particle Size			Residual Drug Content		
	Storage Conditions	0 Day	15 Days	30 Days	0 Day	15 Days	30 Days
RO-EA NTS	4.0 ± 1 °C	105 ± 2.1	108 ± 4.5	107 ± 1.3	99.4 ± 0.11	97.4 ± 0.61	95.5 ± 0.31
	25 ± 2 °C	104 ± 1.1	107 ± 3.5	106 ± 3.2	98.4 ± 0.81	97.4 ± 0.41	96.2 ± 0.25
RO-EA NTS IN SITU GEL	4.0 ± 1 °C	109 ± 6.1	108 ± 1.1	108 ± 5.1	99.1 ± 0.31	98.4 ± 0.21	99.3 ± 0.71
	25 ± 2 °C	109 ± 2.6	109 ± 4.1	106 ± 4.1	99.4 ± 0.31	99.5 ± 0.11	99.1 ± 0.71

## References

[1] Warnakulasuriya S. (2009). Global epidemiology of oral and oropharyngeal cancer. Oral Oncol..

[2] Hirano M., Matsuoka H., Kuroiwa Y., Sato K., Tanaka S., Yoshida T. (1992). Dysphagia following various degrees of surgical resection for oral cancer. Ann. Otol. Rhinol. Laryngol..

[3] Doktorovova S., Souto E.B. (2009). Nanostructured lipid carrier-based hydrogel formulations for drug delivery: A comprehensive review. Expert Opin. Drug Deliv..

[4] Feleszko W., Mlynarczuk I., Balkowiec-Iskra E.Z., Czajka A., Switaj T., Stoklosa T., Giermasz A., Jako’bisiak M. (2000). Lovastatin potentiates antitumor activity and attenuates cardiotoxicity of doxorubicin in three tumor models in mice. Clin. Cancer Res..

[5] Rozados V.R., Hinrichsen L.I., Binda M.M., Gervasoni S.I., Matar P., Bonfil R.D., Scharovsky O.G. (2008). Lovastatin enhances the antitumoral and apoptotic activity of doxorubicin in murine tumor models. Oncol. Rep..

[6] Roudier E., Mistafa O., Stenius U. (2006). Statins induce mammalian target of rapamycin (mTOR)-mediated inhibition of Akt signaling and sensitize p53 deficient cells to cytostatic drugs. Mol. Cancer Ther..

[7] Stirewalt D.L., Appelbaum F.R., Willman C.L., Zager R.A., Banker D.E. (2003). Mevastatin can increase toxicity in primary AMLs exposed to standard therapeutic agents, but statin efficacy is not simply associated with ras hotspot mutations or overexpression. Leuk. Res..

[8] Fujiwara K., Tsubaki M., Yamazoe Y., Nishiura S., Kawaguchi T., Ogaki M., Nishinobo M., Shimamoto K., Moriyama K., Nishida S. (2008). Fluvastatin induces apoptosis on human tongue carcinoma cell line HSC-3, Yakugaku Zasshi. J. Pharm. Soc. Jpn..

[9] Cevc G., Blume G. (2003). Biological activity and characteristics of triamcinolone-acetonide formulated with the self-regulating drug carriers, Transfersomes. Biochim. Biophys. Acta.

[10] Aburahma M.H. (2014). Bile salts-containing vesicles: Promising pharmaceutical carriers for oral delivery of poorly water-soluble drugs and peptide/protein-based therapeutics or vaccines. Drug Deliv..

[11] Gupta A., Aggarwal G., Singla S., Arora R. (2012). Transfersomes: A novel vesicular carrier for enhanced transdermal delivery of sertraline: Development, characterization and performance evaluation. Sci. Pharm..

[12] Kumar A., Pathak K., Bali V. (2012). Ultra-adaptable nanovesicular systems: A carrier for systemic delivery of therapeutic agents. Drug Discov. Today.

[13] Krishnamoorthy B., Chellan V.R., Natarajan T.S., Ranganathan H., Siyad A. (2013). Self nano emulsifying drug delivery system (SNEDDS) of Rosuvastatin calcium: Design, formulation, bioavailability and pharmacokinetic evaluation. Colloids Surf. B.

[14] Zeybek N.D., Gulcelik N.E., Kaymaz F.F., Sarisozen C., Vural I., Bodur E., Canpinar H., Usman A., Asan E. (2011). Rosuvastatin induces apoptosis in cultured human papillary thyroid cancer cells. J. Endocrinol..

[15] El Sharkawi F.Z., El Shemy H.A., Khaled H.M. (2014). Possible anticancer activity of rosuvastatine, doxazosin, repaglinide and oxcarbazepin. Asian Pac. J. Cancer Prev..

[16] Brown M., Hart C., Tawadros T., Ramani V., Sangar V., Lau M., Clarke N. (2012). The differential effects of statins on the metastatic behaviour of prostate cancer. Br. J. Cancer.

[17] Dorai T., Aggarwal B.B. (2004). Role of chemopreventive agents in cancer theraphy. Cancer Lett..

[18] Seeram N.P., Adams L.S., Henning S.M., Niu Y., Zhang Y., Nair M.G., Heber D. (2005). In vitro antiproliferative, apoptotic and antioxidant activities of punicalagin, ellagic acid and a total pomegranate tannin extract are enhanced in combination with other polyphenols as found in pomegranate juice. J. Nutr. Biochem..

[19] Nirmal H.B., Bakliwal S.R., Pawar S.P. (2010). In-Situ gel: New trends in Controlled and Sustained Drug Delivery System. Int. J. Pharm Tech Res..

[20] Ito T., Yeo Y., Highley C.B., Bellas E., Benitez C.A., Kohane D.S. (2007). The prevention of peritoneal adhesions by in situ cross-linking hydrogels of hyaluronic acid and cellulose derivatives. Biomaterials.

[21] Taheri A., Atyabi F., Dinarvnd R. (2011). Temperature-responsive and biodegradable PVA: PVP k30: Poloxamer 407 hydrogel for controlled delivery of human growth hormone (hGH). J. Pediatr. Endocrinol. Metab..

[22] Qushawy M., Nasr A., Abd-Alhaseeb M., Swidan S. (2018). Design, optimization and characterization of a transfersomal gel using miconazole nitrate for the treatment of candida skin infections. Pharmaceutics.

[23] Khajeh M. (2009). Application of Box-Behnken design in the optimization of a magnetic nanoparticle procedure for zinc determination in analytical samples by inductively coupled plasma optical emission spectrometry. J. Hazard Mater..

[24] Guo J., Ping Q., Sun G., Jiao C. (2000). Lecithin vesicular carriers for transdermal delivery of cyclosporine. Int. J. Pharm..

[25] El Maghraby G.M.M., Williams A.C., Barry B.W. (1999). Skin delivery from ultradeformable liposomes: Refinement of surfactant concentration. J. Pharm. Pharmacol..

[26] Rimple S., Munish A., Harmanmeet K. (2012). Thiolated pectin nanoparticles: Preparation, characterization and ex vivo corneal permeation study. Carbohydr. Polym..

[27] Bunjes H., KWestesen K., Koch M.H. (1996). Crystallization tendency and polymorphic transitions in triglyceride nanoparticles. Int. J. Pharm..

[28] Bhatia M., Ahuja M. (2015). Psyllium arabinoxylan: Carboxymethylation, characterization and evaluation for nanoparticulate drug delivery. Int. J. Biol. Macromol..

[29] Miller S.C., Donovan M.D. (1982). Effect of poloxamer 407 gel on the miotic activity of pilocarpine nitrate in rabbits. Int. J. Pharm..

[30] Badgujar S.D., Sontakke M.A., Narute D.R., Karmarkar R.R., Tupkar S.V., Barhate S.D. (2010). Formulation and evaluation of sumatriptan succinate nasal in situ gel using fulvic acid as novel permeation enhancer. Int. J. Pharm. Res. Dev..

[31] Dabhi M.R., Nagori S.A., Gohel M.C., Parikh R.K., Sheth N.R. (2010). Formulation development of smart gel periodontal drug delivery system for local delivery of chemotherapeutic agents with application of experimental design. Drug Deliv..

[32] Wamorkar V., Varma M.M., Manjunath S.Y. (2011). Formulation and evaluation of stomach specific in situ gel of metoclopramide using natural, bioodegradable Polymers. Int. J. Res. Pharm. Biomed. Sci..

[33] Zhang X., Liu J., Qiao H., Liu H., Ni J., Zhang W., Shi Y. (2010). Formulation optimization of dihydroartemisinin nanostructured lipid carrier using response surface methodology. Powder Technol..

[34] Chien M.H., Ying T.H., Hsieh Y.S., Chang Y.C., Yeh C.M., Ko J.L., Yang S.F. (2012). Dioscorea nipponica Makino inhibits migration and invasion of human oral cancer HSC-3 cells by transcriptional inhibition of matrix metalloproteinase-2 through modulation of CREB and AP-1 activity. Food Chem. Toxicol..

[35] Lin C.C. (2007). Berberine induces apoptosis in human HSC-3 oral cancer cells via simultaneous activation of the death receptor-mediated and mitochondrial pathway. Anticancer Res..

[36] Joshi R., Kulkarni R. (2018). Chaudhari, In-vitro and Ex-vivo evaluation of Raloxifene hydrochloride delivery using nano-transfersome based formulations. J. Drug Deliv. Sci. Technol..

[37] Patil P., Khairnar G., Naik J. (2015). Preparation and statisticaloptimization of Losartan Potassium loaded nanoparticles using Box behnken factorial design: Microreactor precipitation. Chem. Eng. Res. Des..

[38] Khairnar G., Mokale V., Naik J. (2014). Formulation and development of nateglinide loaded sustained release ethyl cellulose microspheres by O/W solvent emulsification technique. J. Pharm. Innov..

[39] Siepmann F., Hoffmann A., Leclercq B., Carlin B., Siepmann J. (2007). How to adjust desired drug release patterns from ethylcellulose-coated dosage forms. J. Control. Release.

[40] Alkhalidi H.M., Naguib G.H., Kurakula M., Hamed M.T., Attar M.H., Almatrook Z.H., Aldryhim A.Y., Bahmdan R.H., Khallaf R.A., El Sisi A.M. (2018). In vitro and preclinical assessment of factorial design based nanoethosomal transdermal film formulation of mefenamic acid to overcome barriers to its use in relieving pain and inflammation. J. Drug Deliv. Sci. Technol..

[41] Naveen N.R., Gopinath C., Rao D.S. (2017). Design expert supported mathematical optimization of repaglinide gastro retentive floating tablets: In vitro and in vivo evaluation, Future. J. Pharm. Sci..

[42] Chaudharya H., Kohlib K., Kumar V. (2013). Nano-transfersomes as a novel carrier for transdermal delivery. Int. J. Pharm..

[43] Ahad A. (2012). Formulation and optimization of nanotransfersomes using experimental design technique for accentuated transdermal delivery of valsartan. Nanomed. Nanotechnol. Biol. Med..

[44] Madane R.G., Mahajan H.S. (2016). Curcumin-loaded nanostructured lipid carriers (NLCs) for nasal administration: Design, characterization, and in vivo study. Drug Deliv..

[45] Liu Q., Deng R., Li S., Li X., Li K., Kebaituli G., Li X., Liu R. (2017). Ellagic acid protects against neuron damage in ischemic stroke through regulating the ratio of Bcl-2/Bax expression. Appl. Physiol. Nutr. Metab..

[46] Fahmy U.A. (2018). Augmentation of fluvastatin cytotoxicity against prostate carcinoma PC3 cell line utilizing alpha lipoic–ellagic acid nanostructured lipid carrier formula. AAPS PharmSciTech.

